# Reimplantation of a malleable penile prosthesis in a patient with urethral erosion: a case report

**DOI:** 10.11604/pamj.2024.49.131.45236

**Published:** 2024-12-20

**Authors:** Moussaab Rachid, Omar Lazrak, Hamza Rais, Ghassane El Omri, Younes Houry, Abdeljalil Heddat

**Affiliations:** 1 Department of Urology, Cheikh Khalifa International University Hospital, Mohammed VI University of Sciences and Health (UM6SS), Casablanca, Morocco

**Keywords:** Penile prosthesis, erosion, erectile dysfunction, prosthesis implantation, case report

## Abstract

A 58-year-old man, initially treated for erectile dysfunction, presented with erosion of a penile prosthesis through the urethra one year after his surgical intervention. Clinical examination revealed the extrusion of the left penile prosthesis through the urethral meatus, associated with mild local inflammation and a fever of 39°C. The patient underwent manual extraction of the prosthesis, careful and delicate urethral catheterization, and antibiotic therapy. Two months later, he was readmitted for the insertion of a new penile prosthesis in the left corpora cavernosa and was discharged on the second postoperative day without complications. Thus, erosion of a penile prosthesis is a serious complication requiring prompt removal of the prosthesis, effective antibiotic treatment, and proper urethral healing.

## Introduction

The penile prosthesis (PP) has evolved significantly since its development in the 1970s. It continues to be used not only to treat erectile dysfunction (ED) that is refractory to medical treatment but also to treat Peyronie's disease, psychological impotence, and penile fibrosis [1]. Despite a tendency to present fewer complications than inflatable implants, malleable implants are not without risks, particularly prosthesis erosion. This phenomenon is characterized by the exteriorization of the prosthetic components following repeated contact with the tunica albuginea, leading to skin perforation [2]. We report a case of erosion of the malleable penile prosthesis (MPP) in a 58-year-old patient who underwent successful reimplantation.

## Patient and observation

**Patient information:** a 58-year-old patient was admitted to the emergency department with erosion of a penile prosthesis through the urethra. The patient had no previous medical history, except erectile dysfunction refractory to medical treatment, for which he had received a malleable penile prosthesis outside our establishment a year before his admission.

**Figure 1:**
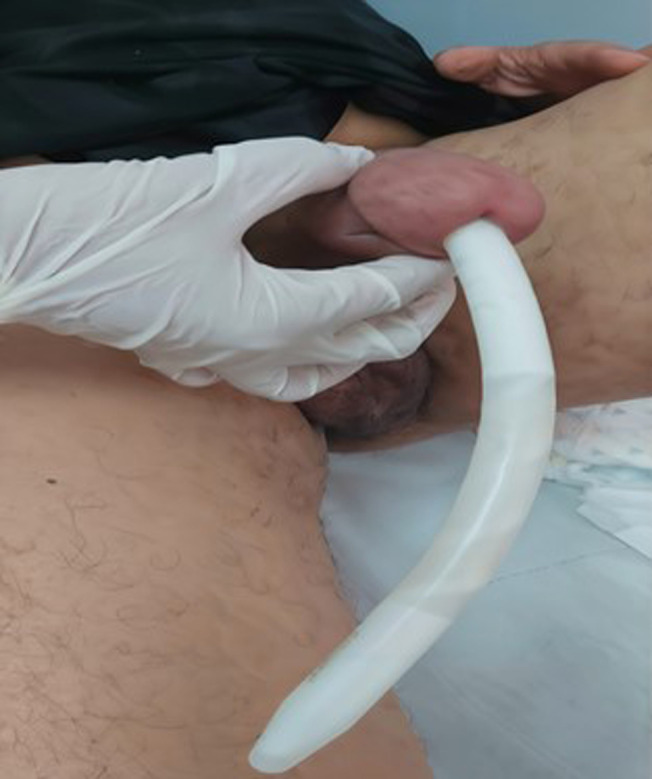
erosion of the left malleable penile prosthesis into the urethra

**Clinical findings:** the patient was conscious, hemodynamically and respiratorily stable on clinical examination, but presented with a fever of 39°C. General and abdominal examination revealed no notable abnormalities. Examination of the external genitalia revealed extrusion of the left penile prosthesis through the urethral meatus, associated with mild local inflammation (Figure 1).

**Timeline:** the patient indicated that he was being treated outside our establishment for erectile dysfunction refractory to medical treatment. He underwent malleable penile prostheses (11 x 18 cm) over a year ago, and immediate post-operative follow-up revealed no abnormalities. Symptoms began 24 hours before admission, with progressive pain in the distal part of the penis, accompanied by erosion and extrusion of the left penile prosthesis through the urethral meatus. Additionally, the patient began to experience dysuria, but without acute urinary retention, urethrorrhagia, or hematuria.

**Diagnostic assessment:** therefore, the diagnosis of a penile prosthesis complicated by extrusion was established.

**Figure 2:**
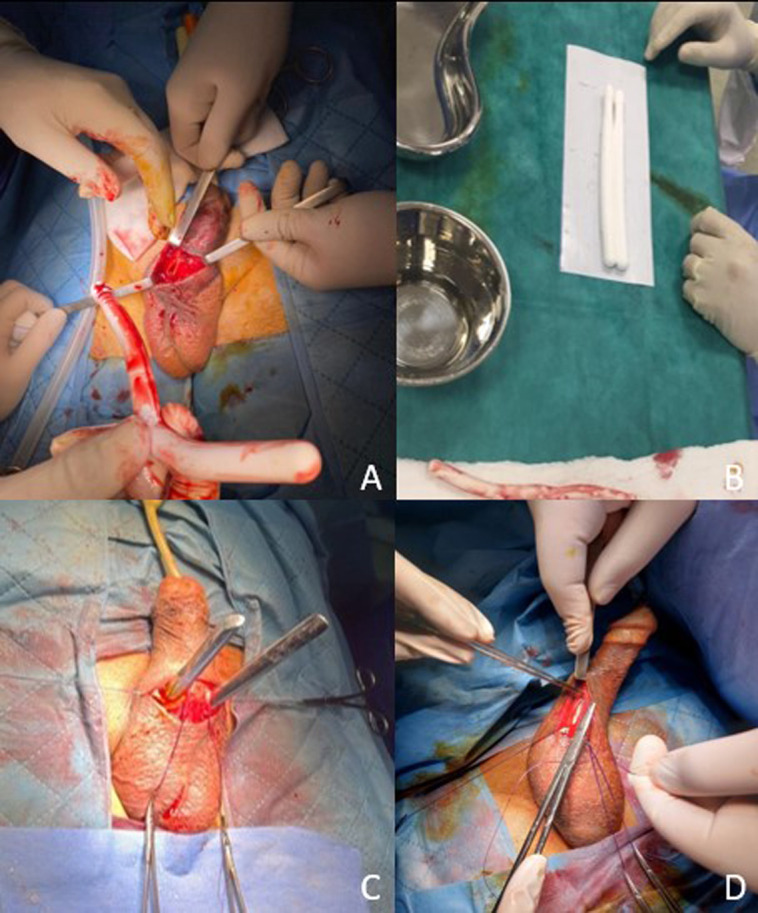
stages of penile prosthesis reimplantation: A) extraction of the right penile prosthesis after incision of the corpora cavernosa; B) choice of new malleable penile prostheses; C) dilation of the corpora cavernosa; D) placement of penile prostheses fitted intra-cavernally

**Figure 3:**
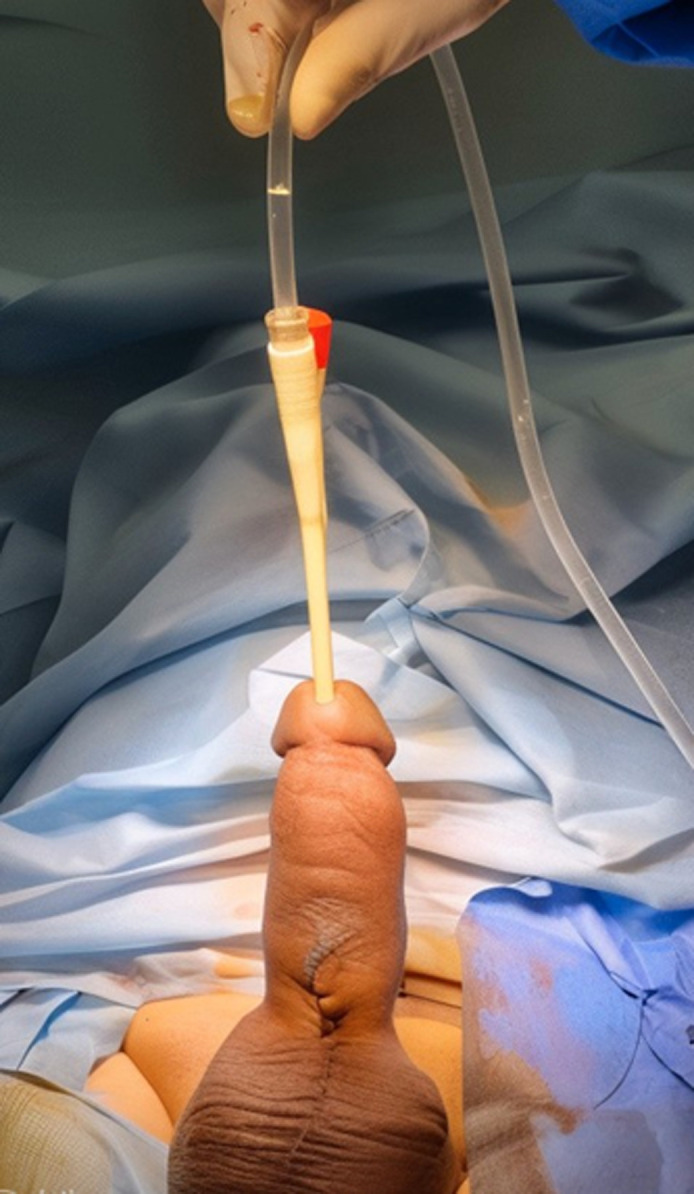
final result after penile prosthesis reimplantation

**Therapeutic intervention:** initially, the patient underwent manual extraction of the prosthesis, careful and delicate urethral catheterization, and dual antibiotic therapy with 3^rd^ generation cephalosporin and gentamicin. His condition gradually improved, with the fever and urinary symptoms disappearing. The patient was hospitalized for seven days. Two months later, the patient was readmitted for the insertion of a new penile prosthesis in the left corpora cavernosa and the replacement of the right prosthesis with a new one. The initial incision was reopened with the release of the envelopes of the penis, followed by dilatation and fitting of a suitable malleable prosthesis (Figure 2). At the end of the operation, the prostheses were in place after skin closure (Figure 3).

**Follow-up and outcomes:** the patient was discharged on the second postoperative day without complication. Recovery was marked by the complete restoration of erectile function and the resolution of pain. The patient was assessed at three and six months postoperatively, with clinical examinations revealing no abnormalities.

**Patient perspective: “**Experiencing the extrusion of my penile prosthesis was alarming and painful, but the prompt and careful treatment I received made a significant difference. The removal, infection management, and subsequent placement of a new prosthesis restored my function and relieved my pain. I am grateful for the attentive and effective care”.

**Patient consent:** informed consent was obtained from the patient for the publication of this article in a journal.

## Discussion

Since their development in the 1970s, penile prostheses have played an increasingly important role in the management of erectile dysfunction refractory to medical treatment. They currently represent the last option in the treatment of erectile dysfunction. Implantation of malleable penile prostheses is a simple procedure with good results, but it is not without complications. Although mechanical complications are rare, they are still possible, such as urethral perforation, hematoma, infection, pain, and erosion. Erosion is an uncommon late complication of malleable penile prosthesis surgery. Erosion generally manifests itself by the protrusion of the prosthesis through the glans, the urethral meatus, or the distal part of the penis [3]. It is most often the result of a progressive weakening of the tunica albuginea [4]. This can be caused by excessive dilation of the corpora cavernosa or prolonged pressure from oversized cylinders [5]. The patients most at risk are those with reduced penile sensitivity, such as diabetics and paraplegics, and those who have undergone several operations on the urethra [6].

The clinical symptoms are dominated by dysuria, urethral discharge, and infection of the prosthesis, which may be complicated by balanitis [3]. In fact, a prosthesis that is oversized in width or length could be responsible for urethral compression, causing ischemia of the urethral wall and thereby promoting erosion. On the other hand, erosion of the penile prosthesis has also been reported by several authors as a complication of urethral catheterization. In a study by Steidle *et al*. [7] of nine patients using clean intermittent catheterization or having an indwelling catheter, the incidence of urethral erosion was high. Although the effective course of action is the removal of the entire device followed by replacement with a MPP, some authors recommend a conservative approach in the absence of infection and necrosis [1]. Additionally, small scrotal erosions can be treated conservatively [8]. Surgery may be reserved for patients with recurrent erosions that do not respond to conservative management. In the reported case, the patient presented with sepsis of urinary origin, so the conservative approach was not indicated. Treatment of erosion depends on the clinical context. One common option is to remove the cylinders and replace the prosthesis after one or two months. Other practitioners opt for a rescue lavage, removing the prosthesis and immediately inserting a malleable prosthesis on the non-eroded parts of the penis [4,5].

## Conclusion

Erosion of the penile prosthesis is a complication that should not be underestimated. All surgeons must be alert to this complication. The prosthesis must be removed and the patient covered with an effective course of antibiotics, as well as ensuring that the urethra has healed properly after four to six weeks. On the other hand, patients should be warned of the risk and should seek medical advice if there is any sign of extrusion, to avoid the risk of imminent erosion.

## References

[1] Basiri A, Zahir M (2023). Successful re-implantation of eroded penile prostheses: Report of two cases and review of the literature. Clin Case Rep.

[2] Cavayero CT, McIntosh GV (2024). Penile Prosthesis Implantation. StatPearls.

[3] Minhas S (2017). Urethral Perforation During Penile Implant Surgery: What to Do. J Sex Med.

[4] Levine LA, Becher EF, Bella AJ, Brant WO, Kohler TS, Martinez-Salamanca JI (2016). Penile Prosthesis Surgery: Current Recommendations From the International Consultation on Sexual Medicine. J Sex Med.

[5] Cayetano-Alcaraz AA, Yassin M, Desai A, Tharakan T, Tsampoukas G, Zurli M (2021). Penile implant surgery-managing complications. Fac Rev.

[6] Kim YD, Yang SO, Lee JK, Jung TY, Shim HB (2008). Usefulness of a malleable penile prosthesis in patients with a spinal cord injury. Int J Urol Off J Jpn Urol Assoc.

[7] Steidle CP, Mulcahy JJ (1989). Erosion of penile prostheses: a complication of urethral catheterization. J Urol.

[8] Talib RA, Shamsodini A, Salem EA, Canguven O, Al Ansari A (2013). Isolated pump erosion of an inflatable penile prosthesis through the scrotum in a diabetic patient. Arch Ital Urol Androl Organo Uff Soc Ital Ecogr Urol E Nefrol.

